# Injuries, Risk Factors, and Prevention Strategies in Bicycle Motocross (BMX): A Scoping Review

**DOI:** 10.1177/19417381241285037

**Published:** 2024-10-26

**Authors:** Claire Rockliff, Karen Pulsifer, Srijal Gupta, Carley B. Jewell, Amanda M. Black

**Affiliations:** † Faculty of Kinesiology, University of Calgary, Canada; ‡ Alberta Children’s Hospital Research Institute, University of Calgary, Canada; § O’Brien Institute for Public Health, University of Calgary, Canada, Department of Community Health Sciences, Cumming School of Medicine, University of Calgary, Canada; ‖ Hotchkiss Brain Institute, University of Calgary, Canada; ¶ Department of Kinesiology, Faculty of Applied Health Sciences, Brock University, Canada

**Keywords:** BMX, injury, prevention, risk factor

## Abstract

**Context:**

Bicycle motocross (BMX) has become increasingly popular since its inclusion in the 2008 Olympics, but it has some of the highest injury rates (IRs) in multisport studies. To support planning for tailored primary prevention, understanding gaps in BMX injury prevention is crucial.

**Objective:**

To examine the evidence on injury incidence, prevalence, risk factors, prevention strategies, and prevention implementation in BMX.

**Data Sources:**

Ovid MEDLINE, Embase, APA PsycInfo, CINAHL, and SPORTDiscus were searched systematically in June 2023.

**Study Selection:**

Articles including BMX and any injury as the main topic or subtopic were searched across multiple databases.

**Study Design:**

A scoping review was designed following the PRISMA Extension for Scoping Reviews (PRISMA-ScR).

**Level of Evidence:**

Level 4.

**Data Extraction:**

BMX injury incidences, prevalence, risk factors, prevention strategies, and prevention implementation were extracted. Two reviewers screened all studies and extracted data independently.

**Results:**

Of the 1856 articles screened, 37 met inclusion criteria. Most studies used injury surveillance at elite competitions or emergency departments, and common injuries were contusions, lacerations, and fractures. IRs provided were based primarily on elite competition and were heterogeneous (eg, 2016 Olympics: 37.5 per 100 athletes; 2007 BMX World Championship: 11.7 per 100 athletes; 1989 BMX Euro Championship: 6.6 per 100 athletes). Only 1 study stratified IRs by BMX discipline (BMX freestyle: IR, 22.2 injuries per 100 athletes; BMX racing: IR, 27.1 per 100 athletes). Few prevention strategies have been evaluated, but reducing the number of riders per race could be helpful.

**Conclusion:**

Most BMX studies do not use recommended injury surveillance methodology. Studies based on emergency department data may underestimate minor injuries and do not adequately measure BMX exposures. Rigorous community-based prospective studies examining IRs for both BMX racing and freestyle, risk factors, and prevention strategies are needed to inform widespread evidence-based prevention strategies.

Bicycle motocross (BMX) emerged in the 1960s as a cost-effective alternative to motorized motocross. BMX bicycles were modified to be lightweight and versatile, suited to both urban and dirt tracks. In 1993, BMX was fully integrated into the Union Cycliste Internationale (UCI) and was officially added to the Olympics in 2008. Today, BMX consists of 2 distinct disciplines: racing and freestyle. Racing involves a sprint-style race that ranges from 300 to 400 meters in distance on either dirt or paved tracks with up to 8 lanes, a gated starting hill, and a combination of berms (ie, banked corners), jumps, and flat sections of varying competition-specific difficulty. In BMX freestyle, riders are judged on their stunt routines.

The UCI noted an increase in BMX participants between 2013 and 2019 at their World Championships; from under 1900 representing 36 countries to over 3700 representing 49 countries.^40,41^ With increased popularity, there is a need to examine BMX injuries as several multisport studies have identified BMX as one of the top sports for injuries.^4,34^ The last BMX injury-specific narrative review was conducted in 1994,^21^ highlighting the need for a comprehensive updated review to inform our understanding of BMX injury prevention and management and to identify key research areas for future studies.

Injury prevention models highlight the importance of injury surveillance to accurately measure injury rates (IRs), understand risk factors and mechanisms, and develop preventative measures tailored to the specific context of the sport.^11^ A comprehensive understanding of these areas is crucial for clinicians and sport administrators to evaluate current and future injury prevention strategies and promote safe sport participation. Scoping reviews are particularly well-suited for describing bodies of literature and identifying gaps in evidence. Moreover, a scoping review can accommodate for the broad range of study designs and reporting heterogeneity in the BMX injury field.^27^ The objectives of this scoping review are 4-fold: to (1) describe studies examining the incidence rates, prevalence, types, and mechanisms of injuries in BMX; (2) explore potential risk factors for BMX injuries; (3) describe prevention strategies; and (4) identify any published models, theories, or data that support successful injury prevention or implementation programs in BMX.

## Methods

### Data Sources and Search Strategy

This review was conducted in alignment with the JBI scoping review guidelines and is reported in accordance with the Preferred Reporting Items for Systematic reviews and Meta-Analyses extension for Scoping Reviews (PRISMA-ScR) guidelines.^39^ A health science librarian provided guidance on the protocol and final search strategy. The search strategy included both keywords and subject headings related to 2 main concepts: (1) Injury AND (2) BMX/bicycle motocross/bike motocross participants and was adapted for use within each database: Ovid MEDLINE (R) In-Process & Other Non-Indexed Citations (OVID, 1946 to present), CINAHL Plus with Full Text (EBSCO, 1982 to present), APA PsycINFO (OVID, 1967 to present), SPORTDiscus with Full Text (EBSCO, 1980 to present), EMBASE (OVID, 1974 to present). The original search strategy used no language or date restrictions or study design filters. The original search was conducted in September 2021, and updated in June 2023. All final search strategies are available in Appendix 1, available in the online version of this article.

### Source of Evidence Selection

Two reviewers independently screened a sample of 100 titles/abstracts in Microsoft Excel to clarify inclusion and exclusion criteria. All search results were uploaded to a screening and data extraction tool (ie, Covidence; Veritas Health Innovation Ltd) for title and abstract screening and full-text eligibility assessment. After duplicates were removed automatically by Covidence, remaining titles and abstracts were reviewed independently by 2 researchers to identify potentially relevant studies. All potentially relevant studies underwent a full-text review by 2 independent reviewers to determine the final study selection. Discrepancies that arose during either title/abstract screening or full-text review were resolved via consensus or consultation with a third reviewer.

To be included, studies were required to meet the following criteria: (1) persons who participate in BMX; and (2) examined injury from BMX participation in one of the following concepts: (a) injury incidence/prevalence/characteristics, (b) risk/protective factors, or (c) prevention/implementation strategies. Non-English studies, editorials, commentaries, or other nonacademic articles were excluded.

### Data Extraction

Data were extracted independently by 2 authors and discrepancies were resolved by consensus. A third reviewer was consulted when necessary. Extraction was completed in a standardized table in Microsoft Excel. Critical appraisal or risk of bias assessment was not completed, per the conducting guidelines of scoping reviews.^27^ The following data were extracted from the included studies: study setting (ie, publication year, design, location of study), sample information (ie, age, sex, level of competition, sample size), definition of injury, definition of exposure, injury characteristics (ie, location, types, severity, mechanism), description of risk factors/prevention strategies and how they were measured, description of implementation factors and how they were measured, and a summary of key results (ie, statistical outcomes and measures of variability within data). Reported risk factors could include both intrinsic (eg, age, sex, gender, weight, height, etc) and extrinsic (eg, weather, racetrack features, competition rules, etc) information. We extracted explored risk factors with effect estimates where possible. The prevention strategies could include but were not limited to changes or differences in rules/regulations/policy, changes to terrain, training strategies, and/or protective equipment. Prevention approaches could include active (ie, behavioral) or passive (ie, environmental) strategies related to education, enforcement, and/or engineering. Implementation factors included strategy adoption, adherence, barriers, and/or facilitators to implementation. For studies evaluating prevention strategies, we extracted or calculated effect estimates where possible (eg, odds ratio, risk ratio, incidence rate ratio).

### Synthesis of the Evidence

Due to the heterogeneity in BMX injury surveillance approaches, results are reported descriptively by study setting and design. When injury incidences, rates, or proportions were not reported (NR) as results within a study, we calculated them using available data. Where possible we have specified the denominator used by presenting a proportion. Injury types and locations were generalized into categories (eg, fractures, sprain/strains, head/face/neck, upper limb, lower limb, etc) that made cross-study comparisons possible. We identified where injury types were NR versus assumed to be zero based on the available information. Where possible, injury locations, types, mechanisms, and severities are provided as a percentage of all BMX injuries within each study. When reporting case studies, we coded the injuries using the Sports Medicine Diagnostic Coding System (SMCDC) and the Orchard Sports Injury and Illness Classification System (OSIIC 13) to be able to comment on any needed modifications specific to BMX.^30^ Since the studies do not present IRs and risk factors homogeneously, and they differ in terms of prevention/implementation strategies and alignment with the goals of our scoping review, we did not conduct any meta-analyses.

## Results

**Figure 1.**
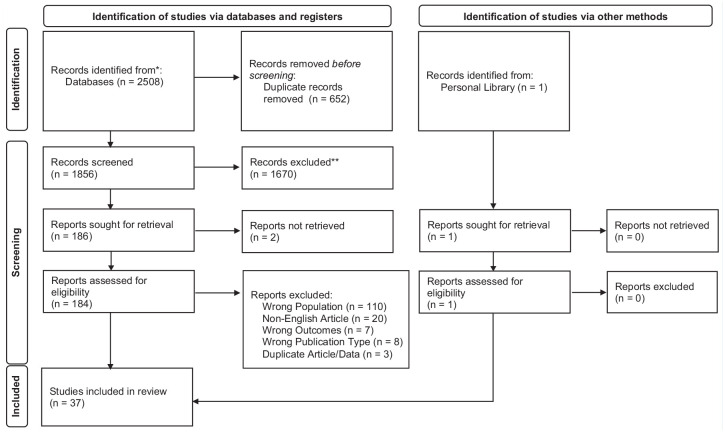
PRISMA flow diagram. PRISMA, preferred reporting items for systematic reviews and meta-analyses.

The database search yielded 2508 records (Figure 1). After completing all stages of the review process, a total of 37 studies were included (see Online Appendix 2 for a full list of included studies).

### Study Characteristics

Table 1 presents the characteristics of studies included in this review. Geographically, most BMX studies were conducted in Europe (37.8%),^1,5,8,9,12,13,16-18,22,31,35,45^ or the United States (24.3%).^14,20,21,23,29,32,38,43,44^ Over one-third of studies (35.1%) were published in the decade after the inclusion of BMX in the 2008 Olympics (see Online Appendix 3 for visual representation of publication trends).^1,3,8,9,14,16,19,24,25,28,29,34,38^ Most studies were cohort (59.5%),^1,3,5,7,9,10,14,17,18,24-26,28,31,33,34,37,38,43-45^ or case studies (27.0%),^8,12,13,19-23,29,36^ and 70.3% of studies were conducted in a hospital setting. Five studies were from national and international championship events.^5,9,25,33,34^ Most BMX studies included athletes who were primarily youth (56.8%). There were no female-athlete-only studies. Many studies did not report the level of competition for BMX athletes (35.1%).

**Table 1.** Study characteristics

Characteristic	Number of Studies (%)
Country/region	
United States	9 (24.3)
Canada	2 (5.4)
Europe	14 (37.8)
Australia	6 (16.2)
New Zealand	1 (2.7)
Brazil	1 (2.7)
Japan	1 (2.7)
Israel	1 (2.7)
Multicountry	1 (2.7)
N/A	1 (2.7)
Publication date	
1980-1989	10 (27.0)
1990-1999	5 (13.5)
2000-2009	2 (5.4)
2010-2019	13 (35.1)
2020-2023	7 (18.9)
Research design	
Review	1 (2.7)
Pre-experimental	1 (2.7)
Cohort	22 (59.5)
Case-control	1 (2.7)
Cross-sectional	2 (5.4)
Case series/case study	10 (27.0)
Setting	
Hospital	26 (70.3)
High school	1 (2.7)
Championship events	5 (13.5)
Local racetracks	2 (5.4)
Other	2 (5.4)
N/A	1 (2.7)
Age	
Youth, ≤18 years	21 (56.8)
Adult, >18 years	7 (18.9)
Both youth and adult	5 (13.5)
NR	3 (8.1)
N/A	1 (2.7)
Sex	
Male	15 (40.5)
Female	0 (0.0)
Both male and female	17 (45.9)
NR	4 (10.8)
N/A	1 (2.7)
Level of competition	
Recreational	12 (32.4)
Competitive/professional	11 (29.7)
NR	13 (35.1)
N/A	1 (2.7)

N/A, not applicable; NR, not reported.

### Injury Incidence and Incidence Rates

All injury definitions broadly encompassed seeking medical attention, except for 1 study that examined self-reported injury.^4^ These studies took place in 3 different settings: championship events, hospitals, and high schools. Injury incidence and incidence rates from championship events are summarized in Table 2. BMX exposure was described as the use of a BMX-specific bike, the participation in designated BMX sport, activities, and/or events, and/or a BMX-specific setting (ie, BMX track and/or park). Only 1 study reported incidence rates with time as the denominator, reporting 1190.48 injuries per 1000 competition hours at the BMX Euro Championships.^5^ The rate of injury at the 2016 Olympics (37.5 [95% CI, 20.2-54.8] per 100 registered athletes) is greater than the rate reported at the 1989 European Championships (6.3 [calculated] per 100 registered athletes).^5,34^ At the most recent 2020 Olympics, IRs for BMX freestyle (22.2 [95% CI, 0.4-44.0] per 100 registered athletes) and racing (27.1 [95% CI, 12.4-41.8] per 100 registered athletes) were reported separately.^33^ Only the 3 studies conducted at the Olympic games reported injuries that occurred in competition versus training. When compared with all other Olympic sports, BMX racing and freestyle injury incidence were reported as highest and second highest in 2020, respectively, whereas overall BMX injury incidence was reported as highest in 2016 and in the top 4 sports in 2012.^9,33,34^

**Table 2.** Incidence rates

Lead Author (Date)	Event (year)	Total BMX Participants	Number of BMX Injuries	Injury Rate	Injuries in Competition	Injuries in Training
Soligard (2023)^33^	Olympics (2020)	66 BMX freestyle: 18 BMX racing: 48	17 BMX freestyle: 4 BMX racing: 13	BMX overall: 25.8^*a*^ BMX freestyle: 22.2 (95% CI, 0.4-44.0) per 100 athletes BMX racing: 27.1 (95% CI, 12.4-41.8) per 100 athletes	9 BMX freestyle: 2 BMX racing: 7	8 BMX freestyle: 2 BMX racing: 6
Soligard (2017)^34^	Olympics (2016)	48	18	37.5 (95% CI, 20.2-54.8) per 100 athletes	14	4
Engebretsen (2013)^9^	Olympics (2012)	48	15	31.3 per 100 athletes^*a*^	11	15
Konczak (2010)^25^	BMX World Championships (2007)	1954	229	11.7 per 100 athletes^*a*^	NR	NR
Brøgger-Jensen (1990)^5^	BMX Euro Championships (1989)	976	61	1190.48 per 1000 competition hours 6.3 per 100 athletes^*a*^	NR	NR

NR, not reported.

a Calculated rate per 100 participants.

Four studies in hospital settings reported the total population served over their study period. The proportion varied with BMX injury representing between 0.5% and 14.3% of injury cases.^7^ BMX injury was also explored in the hospital setting as a proportion of total bicycle injuries. Proportions of BMX injuries reported included 41.9% (119/284),^26^ 44% (131/300),^17^ 49.5% (125/253),^45^ and 74% (212/288) of all bicycle injuries receiving medical attention.^31^ BMX injuries represented 3.2% (6/186) of bicycle injuries requiring admission >24 hours,^3^ as well as 5.8% (6/104) and 10.4% (139/1342) of all cycling-related fractures,^1,10^ 17.2% (5/29) of bicycle riders requiring penile revascularization,^14^ 22.9 % (8/35) of all visceral injuries occurring from bicycle-related injuries,^28^ and 91.6% (121/132) of bicycle-related blunt abdominal trauma.^24^ Among the high school population, only 1 study relied on self-reported injuries from the past year, revealing a BMX IR of 19.4 injuries per 100 students per year (95% CI, 8.91, 37.31).^4^ Several studies explored BMX injuries using varied denominators such as injuries over 40 days or 5 weeks,^18,35^ skatepark injuries,^43^ and children who were injured riding BMX bicycles.^32^

### Injury Type

Nine studies provided details regarding injury types (Table 3). The injury types reported most were minor injuries including contusions and hematomas (34%; range, 6%-43%), lacerations, abrasions, and open wounds (22%; range, 0%-43%), and fractures (18%; range, 0%-35%). One study was not included in Table 3 as it reported injury type only for the more serious injuries that required hospital admission.^31^ Of 212 BMX injuries, the more serious injury types that required admission were head injuries (5.2%, 11/212), fractures ( 4.7%, 10/212), and other (ie, perineal) injuries (0.9%, 2/212).^31^ Three studies investigated specific injury types, and we extracted the following BMX-related proportions: acute sport-related fractures (0.6%, 6/990),^1^ blunt penile vascular injuries (11.1% 5/45),^14^ and severe abdominal injuries from bicycle accidents (22.9%, 8/35).^28^

**Table 3.** Injury type

Lead Author (Date)	Setting	Total BMX Injuries (%)	Lacerations/Abrasions/Open wounds (%)	Contusion/Hematoma (%)	Fractures (%)	Sprains/Strains (%)	Tendinosis/Tendinopathy/Tendon rupture (%)	Dislocations (%)	Visceral Injury (%)	Head injury/Concussion (%)	Dental Injury (%)	Other (%)	NR (%)
Brøgger-Jensen (1990)^5^	Championships	61	26 (43)	18 (30)	4 (7)	8 (13)	NR (0)	NR (0)	NR (0)	2 (3)	1 (2)	2 (3)	0^*a*^ (0)
Engebretsen (2013)^9^	Olympics	15	2 (13)	5 (33)	0 (0)	4 (27)	1 (7)	1 (7)	0^*a*^ (0)	1 (7)	0 (0)	1 (7)	0 (0)
Soligard (2017)^34^	Olympics	18	5 (28)	4 (22)	1 (6)	1 (6)	3 (17)	2 (11)	NR (0)	1 (6)	0 (0)	1 (6)	0 (0)
Soligard (2023)^33^	Olympics	17	6 (35)	1 (6)	3 (18)	3 (18)	2 (12)	0 (0)	NR (0)	1 (6)	0 (0)	1 (6)	0 (0)
Illingworth (1984)^18^	Hospital	100	25 (25)	43^*c*^ (43)	14 (14)	5 (5)	0^*a*^ (0)	0^*a*^ (0)	0^*a*^ (0)	7 (7)	2 (2)	4 (4)	0^*a*^ (0)
Illingworth (1985)^17^	Hospital	135^*b*^	30 (22)	47^*c*^ (35)	25 (19)	3 (2)	NR (0)	NR (0)	NR (0)	25 (19)	5 (4)	NR (0)	NR (0)
Soysa (1984)^35^	Hospital	23	9 (39)	6 (26)	3 (13)	2 (9)	0^*a*^ (0)	0^*a*^ (0)	1 (4)	0 (0)	2 (9)	0^*a*^ (0)	0^*a*^ (0)
Stathakis (1997)^37^	Hospital	254^*b*^	43 (17)	84 (33)	61 (24)	33^*d*^ (13)	NR (0)	NR (0)	NR (0)	13 (5)	NR (0)	NR (0)	20 (8)
Wetterhall (1988)^44^	Hospital	23	0 (0)	9 (39)	8 (35)	5 (22)	0^*a*^ (0)	1 (4)	0^*a*^ (0)	0^*a*^ (0)	0^*a*^ (0)	0^*a*^ (0)	0^*a*^ (0)
	TOTAL	646	146 (22)	217 (34)	119 (18)	64 (10)	6 (1)	4 (1)	1 (0)	50 (8)	10 (2)	9 (1)	20 (3)
	Minimum	0	0 (0)	1 (6)	0 (0)	1 (2)	0 (0)	0 (0)	0 (0)	0 (0)	0 (0)	0 (0)	0 (0)
	Maximum	0	43 (43)	84 (43)	61 (35)	33 (27)	3 (17)	2 (11)	1 (4)	25 (19)	5 (9)	4 (7)	20 (8)

NR, not reported.

a Assumed value.

b Listed multiple injuries per person.

c Includes abrasions.

d Includes strains.

### Injury Location

Ten studies reported injury locations (Table 4). The 3 most common injury locations were upper limbs (40%; range, 26%-67%), head, face, and neck (22%; range, 0%-40%), and lower limbs (21%; range, 18%-40%).

**Table 4.** Injury location

Lead Author (Date)	Setting	Total BMX Injuries (%)	Head/Face/Neck (%)	Trunk/Abdomen (%)	Upper Limb (%)	Lower Limb (%)	Groin (%)	Other (%)	NR (%)
Aitken (2014)^1^	Hospital	6	0 (0)	0 (0)	4 (67)	2 (33)	0 (0)	0 (0)	0 (0)
Fancourt (2022)^10^	Hospital	139	28 (20)	12 (9)	62 (45)	19 (14)	1 (1)	0 (0)	17 (12)
Park (1986)^31^	Hospital	212	85 (40)	NR (0)	55 (26)	53 (25)	2 (1)	NR (0)	17 (8)
Soysa (1984)^35^	Hospital	23	5 (22)	1 (4)	12 (52)	5 (22)	0 (0)	0 (0)	0 (0)
Stathakis (1997)^37^	Hospital	254	56 (22)	23 (9)	122 (48)	46 (18)	NR (0)	8 (3)	NR (0)
Wetterhall (1988)^44^	Hospital	23	2 (9)	1 (4)	14 (61)	6 (26)	0 (0)	0 (0)	0 (0)
Engebretsen (2013)^9^	Olympics	15	1 (7)	1 (7)	6 (40)	6 (40)	0 (0)	1 (7)	0 (0)
Soligard (2017)^34^	Olympics	18	2 (11)	1 (6)	8 (44)	7 (39)	0 (0)	0 (0)	0 (0)
Soligard (2023)^33^	Olympics	17	2 (12)	2 (12)	9 (53)	4 (24)	0 (0)	0 (0)	0 (0)
Konczak (2010)^25^	Championships	131	NR (0)	56 (43)	47 (36)	28 (21)	NR (0)	NR (0)	NR (0)
	TOTAL	838	181 (22)	97 (12)	339 (40)	176 (21)	3 (0.3)	9 (1)	34 (4)
	Minimum		0 (0)	0 (0)	4 (26)	2 (14)	0 (0)	0 (0)	0 (0)
	Maximum		85 (40)	56 (43)	122 (67)	53 (40)	2 (1)	8 (7)	17 (12)

NR, not reported.

Two studies dichotomized injury location as above or below the neck in a hospital setting.^18^ These studies reported injury distributions of 32% and 19% above the neck and 68% and 78% below the neck, respectively.^17,18^ In addition, 3 studies focused on specific injury locations, and we extracted the proportions of injuries related to BMX involvement in these areas. These specific locations included male genitals (11.1%, 5/45),^14^ the abdomen (e.g., abdominal wall hematoma, kidney, and spleen) (22.9%, 8/35),^24,28^ and facial regions (14.3%, 38/265).^7^

### Injury Severity

Three studies conducted at the Olympic games reported severity as time lost from sport due to injury.^9,33,34^ Most recently at the 2020 Olympics, BMX racing and BMX freestyle injuries resulted in time loss ≥1 day (14.6% [7/48] and 5.6% [1/18]) and >7 days time loss (10.4% [5/48] and 5.6% [1/18]).^33^ At the 2016 Olympics, 50% (9/18) of BMX injuries resulted in time loss ≥1 day, and 27.8% (5/18) resulted in >7 days time loss. BMX had the highest proportion (10%, 5/48) of injuries that resulted in a time loss of >7 days at the 2016 games.^34^ Earlier at the 2012 Olympics, 33.3% (5/15) BMX injuries resulted in time loss ≥1 day, and 13.3% (2/15) resulted in >7 days time loss. During these games, BMX had the third highest rate (4%, 2/48) of injury that involved >7 days time loss.^9^ The 1989 BMX European Championship reported that 52.5% (calculated 32/61) of injuries required medical attention beyond first aid, 16.7% (calculated 10/61) were examined at a hospital, and 3.3% (calculated 2/61) were admitted to a hospital.^9^

Four studies conducted in hospital emergency departments in the 1980s reported BMX injuries on 3- to 5-point grade scales; however, inconsistent definitions of grades and varying grade scales make it difficult to compare BMX injury severity in these settings.^17,18,31,45^ Four cohort studies conducted in emergency departments did provide some insight into severity by reporting the proportion of BMX injuries that required hospital admission: 4% (1/23),^35^ 6.9% (9/131),^17^ 9% (9/100),^18^ and 18% (calculated 36/199).^37^ One study explored cycling-specific fractures and reported severity as injuries that required surgery, where BMX injuries comprised 8.7% (73/837).^10^

Four studies investigated only injuries that resulted in hospital admission. Specifically, Beck et al^3^ investigated bike injuries resulting in hospital admission for >24 hours, Klin et al^29^ and Muthucumaru et al^33^ investigated abdominal injuries that required admission, and Goldstein and Bastuba^14^ investigated penile injuries that required admission.

### Injury Mechanism

Four studies in hospital settings reported that all of the BMX injuries were a result of blunt force trauma (*n* = 5),^14^ (*n* = 8),^28^ (*n* = 19),^32^ and (*n* = 121).^24^ The proportions of BMX injuries due to falls and collisions were reported in 2 studies, where 48% (136/284),^26^ and 79% (157/199) resulted from falls,^37^ and 41% (calculated 116/284) and 12.5% (25/199) resulted from collisions.^26,37^ Handlebars were a subcategory of blunt force trauma or falls, identified as a common contributing factor to injuries in hospital settings. Three studies described proportions of injuries that resulted from falls over the handlebars: 5% (10/199),^37^ 18% (23/131),^17^ and 23% (23/100),^18^ whereas 1 study reported 100% (8/8) of injuries were attributed to handlebar impact.^28^ Stunts were also a common contributing factor, with proportions reported over a vast range of: 0.5% (1/199),^37^ 37% (46/125),^45^ 39% (51/131),^17^ 40% (40/100),^18^ 43% (10/23),^35^ and 100% (8/8).^28^

### Risk Factors

Very few studies provided estimated risk ratios (RR) or odds ratios (OR) to examine risk factors. The risk factor investigated most frequently was sex, with inconsistent results. At the 2020 Olympic games, there was no overall difference in risk ratio between men and women for BMX injuries (RR = 0.93 [0.83-1.06]).^33^ Engebretsen et al^9^ and Soligard et al^36^ found no significant differences between sex and incidence of injury, whereas Illingworth^18^ found no significant differences between sex and severity of injury. Brøgger-Jensen et al^5^ and Konczak^25^ observed a greater rate of injury in female riders but did not report statistical significance. One study reported that male BMX participants are more likely to incur facial fractures than female participants (95% CI, 0.981 [0.437-2.203]).^7^

Three studies investigated age and found no correlation between incidence,^5,17^ or severity of injury^18^; however, for BMX-related fractures, over half (55.3% calculated) of injuries occurred in children aged 11 to 16 years.^7^

At the 2016 Olympic games, BMX injury sustained during competition was significantly higher than in training (RR = 3.50 [1.15-10.63]).^34^

One study examined the relationship between the severity of abdominal injury (ie, BMX riders represented 91.6% of the bicycle sample) and impact from either the handlebars or a fall.^24^ More specifically, handlebar impact demonstrated significant increases in admissions to intensive care (28.4% handlebar vs 23.26% falls; *P* = 0.05) and surgical procedures (7.86% handlebar vs 2.33% falls; *P* = 0.02). No significant correlation was reported between a history of previous injury in relation to the severity of new injury.^18^

### Prevention and Implementation Strategies

Three studies evaluated prevention strategies. Hurst et al^16^ examined the use of helmets and neck braces to reduce the number and magnitude of translational and rotational accelerations. Results show that the number of above threshold accelerations observed at the head were significantly reduced when riders wore a neck brace,^16^ but this was not evaluated for its effect on IRs. The second study examined IRs before and after the number of riders in each race was reduced from 6 to 4. Results showed that when the number of riders per race was reduced, the IRs decreased from 2.1 injuries per 1000 BMX participants to 1.6 injuries per 1000 BMX participants, but the change was not significantly significant.^44^ Hardwicke et al^15^ examined competitive cyclists’ perceptions of helmet use as a concussion prevention strategy. Although 95.1% of all cyclists would wear a helmet during competition even if not required, BMX cyclists were more likely to correctly report that helmets do not fully prevent concussions as opposed to other disciplines and were less likely to seek medical care compared with other cyclists (ie, cyclo-cross) after a crash resulting in contact with the floor.^15^

### Case Studies

Table 5 summarizes injury types and locations as well as the most suitable SMDCS and OSIIC 13 codes for each injury. All case studies examined men, aged 14 to 26 years, with BMX injuries. Injury severity in case studies was inconsistently reported; however, 3 cases of catastrophic injury were noted, 2 thoracolumbar spine fractures resulted in associated paraplegia,^22^ and 1 neck fracture resulted in permanent quadriplegic paralysis.^21^

**Table 5.** Case study injuries

Lead Author (date)	Location	Type	SMDCS Code	SMDCS Description	OSIIC 13 Code	OSIIC 13 Diagnosis
Durand (2018)^8^	Hip	Dislocation,^*b*^ fracture of the femoral head, avascular necrosis	HI.90.00 HI.34.13 HI.71.45	Other hip injury; Femoral neck/head fracture	GZX GFN GVH	Hip/groin pain not specified; fractured neck of femur; hip arterial injury/disorder, vascular injury hip joint
Fixsen (1989)^12^	Elbows	Loose bodies, fracture	EL.43.21 EL.34.20	Elbow Loose body; Lateral epicondyle growth plate / physeal injury	ECL EFX	Loose body in elbow; elbow fractures
Gaskell (1987)^13^	Lumbar spine	Disc protrusion L5/S1, flattening L5 nerve root, impinging upon S1	LS.22.39	Lumbar nerve root impingement	LNA	Lumbosacral nerve root impingement due to foraminal stenosis bony and disc
Ipaktchi (2010)^19^	Neck	Subclavian artery and jugular vein rupture,^*b*^ fractured rib	NE.90.00 CH.32.13	Other neck injury; Rib fracture	NZ1 CF1	Neck pain undiagnosed; fractured rib(s)
Izumi (2006)^20^	Groin	Hematuria, ecchymosis, torn urethra and extravasation. Testicular calcification	HI.80.46 HI.60.25	Testicular contusion; Hip laceration	GO2 GKX	Testicular/scrotal contusion/hematoma; hip and groin laceration
Jackson (1994)^21^	Neck^*a*^	Fracture	NE.31.13	Cervical spine fracture	NFC	Cervical fracture/s
Johnson (1987)^22^	Thoracolumbar spine^*a*^	Fracture-dislocation (2 cases)	TS.31.13	Thoracic spine fracture	DFX	Thoracic spine fracture
Kirchberg (2021)^23^	Thoracic spine	Compression fracture	TS.31.13	Thoracic spine fracture	DFX	Thoracic spine fracture
Oller (2012)^29^	(Illness) cardiovascular	Supraventricular tachycardia	CV.01.83	Other cardiovascular illness/disorder	MCZX	Other cardiovascular disease
Sparnon (1982)^36^	Groin	Avulsion, laceration (2 cases)	HI.60.25	Hip laceration	GKX	Hip and groin laceration

a Catastrophic injury.

b Most suitable code represented.

## Discussion

This scoping review highlights key findings and gaps in the context of BMX regarding IRs, characteristics, risk factors, prevention strategies, and implementation (Table 6). Injury rates at tournaments with BMX events ranged between 6.3 and 37.5 per 100 athletes.^5,9,33,34^ Comparatively, in hospital settings, the proportion of BMX injuries that received medical attention ranged from 44% to 74%. However, most hospital studies reporting BMX injury did not have a population-based denominator. Exclusively exploring BMX injury incidence outside of hospital settings may provide a more accurate understanding of chronic and/or less serious BMX injury.

**Table 6.** Summary of future research recommendations

Injury characteristics (ie, definition, incidence, rate, classification, mechanism)
• Explore BMX incidence outside of hospital settings. • Classify population by BMX participation level. • Report BMX exposure rates per 100 runs. • Report BMX freestyle injury rates as a rate per number of stunts performed. • Standardized categorization of body regions/body parts. • Precise location categorization for groin, hip/groin, perineum, and/or genital injuries. • Report collisional agent for blunt force injuries where possible. • Description of the direction of fall or collisional agent specific to BMX bike. • Explore reasons why reporting guidelines are inconsistently followed.
Risk factors
• Explore BMX handlebar design elements in prospective cohort designs. • Classify stunts into categories and compare risk factors between stunts to determine modifiability.
Prevention strategies
• Consider context differences between sport and recreational BMX. • Explore nonmandatory equipment (ie, neck braces) prevention implementation context. • Examine the number of riders participating in BMX races.

BMX sport entails racing and freestyle competition; yet this scoping review identified that most studies explore BMX injury in populations (ie, children) that may not have intended to participate in the sport of BMX, but rather simply rode a BMX bike. This highlights a gray area between the definitions of BMX as a sport and merely riding a BMX bike (eg, as a means of transportation). To combat the lack of definition clarity in BMX sport, future research should classify their population both by BMX style and with respect to the level of participation (eg, recreational, competitive, professional, etc).

The review on cycling-specific surveillance methods recommends reporting BMX exposures per 1000 hours^6^; however, only 1 included study followed that recommendation and reported 1190.48 per 1000 hours at the 1989 European Championship.^5^ This denominator resulted in a very high incidence rate, likely because the mean reported race heat time was only 48 seconds. Given that short race times are common in BMX racing and freestyle events, we suggest that, in addition to using 1000 hours as an exposure rate, future studies also report per 100 runs as per the cycling-specific surveillance methods to report IRs.^6^ While this may limit comparability with other sports, we believe this second recommendation is better suited for the quick nature of BMX racing and freestyle competitions. In addition, to support researchers and practitioners in accurately reporting BMX injuries, future research may benefit from exploring reasons why reporting guidelines are inconsistently followed.

For BMX freestyle competitions specifically, athletes’ stunt performance (ie, quantity and quality) is a metric of success and between-athlete comparison. Athletes may consider their current standing compared with competitors when deciding how many and which stunts to perform. It may be advantageous to report IRs in BMX freestyle events as a rate per number of stunts performed; however, the feasibility of this approach may be challenging. The most recent International Olympic Committee (IOC) consensus statement also recommends surveilling time at risk in training versus competition,^2,6^ an element that was not found in this scoping review except for studies evaluating the 2020, 2016, and 2012 Olympic games.^9,33,34^

The top 3 most frequently reported injury locations by studies in this review were upper limbs, head/face/neck, and lower limbs. Given that location reporting standards varied across studies in this review, injury location comparisons were limited to overall body regions (eg, upper limb/lower limb, above/below neck, etc) as opposed to specific body parts (eg, forearm, ankle, wrist, etc). Using a standardized categorization of body regions and/or body parts for injury reporting is warranted for future studies.^2^

In that vein, 3 studies in this review examined erectile dysfunction and testicular injury in BMX athletes.^14,20,36^ However, the reported injury incidence of areas that relate to erectile dysfunction may be underrepresented due to the younger age of the population represented by this review and how injuries are coded. For example, only 1 study in this review reported “groin” as an injury location,^31^ while another grouped “genital laceration” within abdominal injury.^28^ As the BMX bike saddle is a point of primary contact for athletes, future BMX surveillance studies may warrant precise location categorization for the groin, hip/groin, perineum, and/or genital injuries.

Moreover, the IOC injury reporting guidelines for these locations presents an added challenge for BMX-specific reporting. Injury reporting in these locations may be divided among the current “hip/groin” category described as “hip and anterior musculoskeletal structures (eg, pubic symphysis, proximal adductors, iliopsoas)” and “lumbosacral spine” category described as “lumbar spine, sacroiliac joints, sacrum, coccyx, buttocks.”^2^ Specifically, details regarding genitals and perineum are not covered in these descriptions and the injury may fall anatomically between the pubic symphysis and coccyx. Similarly, the cycling-specific consensus highlights a common cycling condition (ie, numbness of the genitals or perineum/pudendal neuropathy/male or female sexual or urinary dysfunction) and recommends the broader classification to be represented in the “lumbosacral spine” location.^6^ Meanwhile, the SMCDC and OSIIC 13 coding systems place testicular trauma in the “hip/groin” category.^30^ Related to the apparent incongruences in injury locations, it appears that, whereas most injuries sustained in published case studies are well represented in the current SMCDC and OSIIC 13 coding systems; injuries such as hip dislocation and neck vascular injury are not well represented. Discussion of recommendations for these classifications at the next update of the International Olympic Committee and UCI consensuses would be warranted.

### Injury Mechanism

Measures of injury mechanism varied by environmental (ie, blunt force trauma, handlebars) and contextual (ie, stunts) factors that were highly heterogeneous. Those persons reporting blunt force injuries should record the collisional agent responsible for the trauma. Some studies categorized “falls” versus “collisions,” but failed to explicitly state that a fall involves contact with the ground. The cycling-specific consensus provides a clearer distinction by categorizing “the ground only” as a collision agent.^6^ “Handlebars” were also referred to as mechanisms of injury; however, some studies described a fall over the handlebars, while others described an impact with the handlebars. BMX-specific surveillance may benefit from describing the direction of a fall (eg, forward over handlebars, sideways, or backward off bike) or a collision agent specific to the BMX bike (eg, handlebars, pedal, saddle). Further, the cycling-specific consensus provides an extensive list of cycling-related mechanisms and circumstances that would be applicable in sporting and bicycle riding contexts (eg, colliding with a car)^6^; however, these may not relate directly to BMX sport contexts given that athletes often compete and train in closed tracks. Thus, articulation of BMX sport-specific injury mechanisms is warranted.

### Risk Factors

Studies examining risk factors in BMX are limited in both number and methodological rigor. Although we aimed to investigate studies that explored intrinsic (eg, age, sex, gender, weight, height, etc) and extrinsic (eg, weather, racetrack features, competition rules, etc) factors, only 1 study explored sex and competition versus training in a univariate analysis reporting risk ratio. Additional risk factors discussed by authors included BMX handlebar design elements (eg, ridged design, 360° rotation, padding).^12,19,24,35,36^ However, these will need to be explored further in future prospective cohort studies accounting for multivariable modeling and exposure. Another risk factor that was not specifically examined in BMX studies but may be relevant to examine further is stunts. Senturia et al^32^ invested stunts as a risk factor for bicycling (not specific to BMX), and reported an estimated odds ratio of 2.6 (95% CI, 0.5-10.5; *P* > 0.05) when compared with nonstunt bicycle riding but were absent for BMX specifically. Classifying stunts into various categories and comparing risk factors between different stunt types may be warranted to understand which stunts may be modifiable.

### Prevention Strategies

Some potential prevention approaches were highlighted in discussions of articles but not evaluated. Specific strategies included adequate supervision, reducing speed,^22^ wearing appropriate protective equipment,^37^ and reducing the number of riders per race.^44^

When considering that BMX sport competitions are reliant on speed and mandate athletes to wear protective equipment, these concerns may pertain more to recreational BMX bicycle riding or introductory BMX sport, and thus future prevention strategy investigations must adequately consider the difference in context between BMX sport and recreational BMX (ie, riding a BMX bike). Moreover, studies that were concerned about the lack of safety equipment may be outdated and more current suggestions of prevention strategies are absent. Sport BMX participants are currently required to wear full-face helmets, long-sleeved jerseys, long pants (or suitable knee and shin protection), and gloves. However, other safety equipment (ie, back, elbow, knee, shoulder, and/or cervical vertebrae) is only recommended.^42^ These nonmandatory pieces of equipment may warrant further investigation as prevention strategies, including additional research to elaborate on the finding that neck braces reduce subconcussive impacts.^38^ There were very few data to inform injury prevention implementation contexts.

## Strengths and Limitations

This scoping review provides a broad overview of injury characteristics, risk factors, and prevention strategies in the literature pertaining to BMX injury, highlighting the availability of studies, and reporting standards related to the Translating Research into Injury Prevention Practice framework. Our scoping review approach allowed us to summarize and synthesize the current state of BMX sport injury literature as it relates to injury characteristics (ie, incidence, types, mechanisms, etc), BMX injury risk factors, and current prevention strategies, which may be useful for future planning of BMX injury surveillance programs, intervention development, and relevant policymakers. However, the review has major limitations. Methodologically, scoping reviews do not assess the quality of individual studies, which is problematic when assessing and comparing the rigor of specific studies or groups of studies. For instance, given that most studies defined injury as medical treatment received at either sporting events or hospitals, the results of this scoping review may be biased toward the incidence of sudden onset, acute injuries that require medical care.

Similarly, it is more than likely that we missed available data on BMX athletes. This may be due to excluding gray literature from the search strategy and excluding studies published in languages other than English. Although we can comment on the lack of studies that use recommended surveillance strategies, in alignment with scoping review methodology we did not evaluate the risk of bias of the included studies, and thus cannot directly comment on low quality research.

Moreover, BMX is often included under the larger umbrellas of all bicycling activities or all skatepark activities. We excluded studies regarding injury incidence rates, proportions, characteristics, and risk factors if we were unable to extract BMX-specific data from generalized bicycling or skatepark data. Reported injury incidence may also be limited due to inconsistencies between studies where multiple injuries were sustained from a single injury event. Some studies reported only the most serious injury, while others tracked and reported multiple injuries per event or athlete.

## Conclusion

While BMX is gaining popularity, most BMX studies do not use appropriate injury surveillance methodology. Studies based on emergency room data may underestimate minor injuries and do not provide adequate measures of BMX exposure. Reducing the number of riders per race may be a modifiable risk factor that requires further examination. More rigorous community-based prospective studies examining IRs, risk factors, and prevention strategies in both BMX freestyle and racing are needed to inform widespread evidence-based prevention strategies.

## Supplemental Material

sj-pdf-1-sph-10.1177_19417381241285037 – Supplemental material for Injuries, Risk Factors, and Prevention Strategies in Bicycle Motocross (BMX): A Scoping Review Supplemental material, sj-pdf-1-sph-10.1177_19417381241285037 for Injuries, Risk Factors, and Prevention Strategies in Bicycle Motocross (BMX): A Scoping Review by Claire Rockliff, Karen Pulsifer, Srijal Gupta, Carley B. Jewell and Amanda M. Black in Sports Health

sj-pdf-2-sph-10.1177_19417381241285037 – Supplemental material for Injuries, Risk Factors, and Prevention Strategies in Bicycle Motocross (BMX): A Scoping Review Supplemental material, sj-pdf-2-sph-10.1177_19417381241285037 for Injuries, Risk Factors, and Prevention Strategies in Bicycle Motocross (BMX): A Scoping Review by Claire Rockliff, Karen Pulsifer, Srijal Gupta, Carley B. Jewell and Amanda M. Black in Sports Health

sj-pdf-3-sph-10.1177_19417381241285037 – Supplemental material for Injuries, Risk Factors, and Prevention Strategies in Bicycle Motocross (BMX): A Scoping Review Supplemental material, sj-pdf-3-sph-10.1177_19417381241285037 for Injuries, Risk Factors, and Prevention Strategies in Bicycle Motocross (BMX): A Scoping Review by Claire Rockliff, Karen Pulsifer, Srijal Gupta, Carley B. Jewell and Amanda M. Black in Sports Health
