# Supplementary material for: Injuries, Risk Factors, and Prevention Strategies in Bicycle Motocross (BMX): A Scoping Review
Source: Sports Health. 2024 Oct 26;17(5):965–77. doi: 10.1177/19417381241285037 (PMC11556568; doi:10.1177/19417381241285037)
Supplement: sj-pdf-1-sph-10.1177_19417381241285037 – Supplemental material for Injuries, Risk Factors, and Prevention Strategies in Bicycle Motocross (BMX): A Scoping Review [file sj-pdf-1-sph-10.1177_19417381241285037.pdf]

Appendix 1. Final Search Strategy

Database(s): **Ovid MEDLINE(R) and Epub Ahead of Print, In-Process, In-Data-Review & Other Non-Indexed Citations and Daily** 1946 to June 22, 2023
Search Strategy:

#	Searches
1	injur*.mp.
2	accident*.mp.
3	trauma*.mp.
4	Accidents/
5	exp Athletic Injuries/
6	exp Brain Concussion/
7	concuss*.mp.
8	exp "Wounds and Injuries"/
9	wound*.mp.
10	exp Fractures, Bone/
11	fracture*.mp.
12	exp Pain/
13	pain.mp.
14	dislocat*.mp.
15	exp "Sprains and Strains"/
16	(sprain or sprains).mp.
17	exp Tears/
18	(tear or tears).mp.
19	abrasion*.mp.

20	(cut or cuts).mp.
21	exp Lacerations/
22	laceration*.mp.
23	exp Burns/
24	(burn or burns).mp.
25	scrape*.mp.
26	exp Brain Injuries, Traumatic/
27	"MTBI".mp.
28	"TBI".mp.
29	1 or 2 or 3 or 4 or 5 or 6 or 7 or 8 or 9 or 10 or 11 or 12 or 13 or 14 or 15 or 16 or 17 or 18 or 19 or 20 or 21 or 22 or 23 or 24 or 25 or 26 or 27 or 28
30	BMX.mp.
31	((track or "off-road" or freestyl* or competitive* or terrain or skatepark*) adj5 (cycling or bicycl* or cyclist* or rider* or racer*)).mp.
32	(motocross or "moto-cross" or supercross).mp.
33	30 or 31 or 32
34	29 and 33

Database(s): **Embase** 1974 to 2023 June 23
Search Strategy:

#	Searches
1	injur*.mp.
2	accident*.mp.
3	trauma*.mp.

4	exp accident/
5	exp sport injury/
6	exp brain concussion/
7	concuss*.mp.
8	exp wound/
9	exp injury/
10	wound*.mp.
11	exp fracture/
12	fracture*.mp.
13	exp pain/
14	pain.mp.
15	dislocat*.mp.
16	exp sprain/
17	(sprain or sprains).mp.
18	(tear or tears).mp.
19	abrasion*.mp.
20	(cut or cuts).mp.
21	exp laceration/
22	laceration*.mp.
23	exp burn/
24	(burn or burns).mp.
25	scrape*.mp.

26	exp traumatic brain injury/
27	"MTBI".mp.
28	"TBI".mp.
29	1 or 2 or 3 or 4 or 5 or 6 or 7 or 8 or 9 or 10 or 11 or 12 or 13 or 14 or 15 or 16 or 17 or 18 or 19 or 20 or 21 or 22 or 23 or 24 or 25 or 26 or 27 or 28
30	BMX.mp.
31	((track or "off-road" or freestyl* or competitive* or terrain or skatepark*) adj5 (cycling or bicycl* or cyclist* or rider* or racer*)).mp.
32	(motocross or "moto-cross" or supercross).mp.
33	30 or 31 or 32
34	29 and 33

Database(s): **APA PsycInfo** 1806 to June Week 3 2023
Search Strategy:

#	Searches
1	injur*.mp.
2	exp Injuries/
3	Accident*.mp.
4	Accidents/
5	Trauma*.mp.
6	exp Brain Concussion/
7	concuss*.mp.
8	exp Wounds/
9	wound*.mp.

10	fracture*.mp.
11	exp Pain/
12	pain.mp.
13	dislocat*.mp.
14	(sprain or sprains).mp.
15	(tear or tears).mp.
16	abrasion*.mp.
17	(cut or cuts).mp.
18	laceration*.mp.
19	exp Burns/
20	(burn or burns).mp.
21	scrape*.mp.
22	exp Traumatic Brain Injury/
23	"MTBI".mp.
24	"TBI".mp.
25	1 or 2 or 3 or 4 or 5 or 6 or 7 or 8 or 9 or 10 or 11 or 12 or 13 or 14 or 15 or 16 or 17 or 18 or 19 or 20 or 21 or 22 or 23 or 24
26	BMX.mp.
27	((track or "off-road" or freestyl* or competitive* or terrain or skatepark*) adj5 (cycling or bicycl* or cyclist* or rider* or racer*)).mp.
28	(motocross or "moto-cross" or supercross).mp.
29	26 or 27 or 28
30	25 and 29

CINAHL Plus with Full Text (Ebsco)
Search modes - Find all my search terms

#	Query
S1	(MH "Accidents")
S2	(MH "Trauma")
S3	accident*
S4	trauma*
S5	"injur*"
S6	"dislocat*"
S7	sprain or sprains
S8	tear or tears
S9	"abrasion*"
S10	cut or cuts
S11	(MH "Tears and Lacerations+")
S12	"laceration*"
S13	(MH "Burns+")
S14	(MH "Athletic Injuries+") OR (MH "Cycling Injuries")
S15	burn or burns
S16	"scrape*"
S17	(MH "Brain Injuries+")
S18	"MTBI"
S19	"TBI"
S20	(MH "Brain Concussion+")
S21	"concuss*"

S22	(MH "Wounds and Injuries+")
S23	"wound*"
S24	"fracture*"
S25	(MH "Pain+")
S26	"pain"
S27	S1 OR S2 OR S3 OR S4 OR S5 OR S6 OR S7 OR S8 OR S9 OR S10 OR S11 OR S12 OR S13 OR S14 OR S15 OR S16 OR S17 OR S18 OR S19 OR S20 OR S21 OR S22 OR S23 OR S24 OR S25 OR S26
S28	"BMX" or motocross or "moto-cross" or supercross
S29	(track or "off-road" or freestyl* or competitive* or terrain or skatepark) N5 (cycling or bicycl* or cyclist* or rider* or racer*)
S30	S28 OR S29
S31	S27 AND S30

SportDiscus with Full Text (Ebsco)
Search modes - Find all my search terms

#	**Query**
S1	DE "SPORTS accidents" OR DE "CYCLING accidents"
S2	accident*
S3	trauma*
S4	injur*
S5	pain
S6	tear or tears
S7	cut or cuts
S8	laceration*

S9	DE "SPORTS injuries" OR DE "CYCLING injuries" OR DE "MOTORCYCLING injuries"
S10	DE "BURNS & scalds"
S11	burn or burns
S12	scrape*
S13	DE "BRAIN injuries" OR DE "BRAIN damage"
S14	mtbi
S15	tbi
S16	DE "BRAIN concussion" OR DE "POSTCONCUSSION syndrome"
S17	concuss*
S18	DE "WOUNDS & injuries" OR DE "BLUNT trauma" OR DE "BURNS & scalds" OR DE "CRASH injuries" OR DE "DISLOCATIONS (Anatomy)" OR DE "HEAD injuries" OR DE "OVEREXERTION injuries" OR DE "OVERUSE injuries" OR DE "PENETRATING wounds" OR DE "PHYSIOLOGIC strain" OR DE "RUPTURE of organs, tissues, etc." OR DE "SOFT tissue injuries" OR DE "SPORTS injuries"
S19	wound*
S20	DE "BONE fractures" OR DE "ANKLE fractures" OR DE "AVULSION fractures" OR DE "CARTILAGE fractures" OR DE "COMMINUTED fractures" OR DE "FACIAL bone fractures" OR DE "FRACTURE healing" OR DE "HEEL bone fractures" OR DE "JONES fracture" OR DE "PELVIC fractures" OR DE "STRESS fractures (Orthopedics)" OR DE "WRIST fractures"
S21	fracture*
S22	DE "PAIN"
S23	(S1 OR S2 OR S3 OR S4 OR S5 OR S6 OR S7 OR S8 OR S9 OR S10 OR S11 OR S12 OR S13 OR S14 OR S15 OR S16 OR S17 OR S18 OR S19 OR S20 OR S21 OR S22)
S24	DE "BICYCLE motocross" OR DE "BMX bikes" OR DE "X Games (Extreme sports)" OR DE "MOTOCROSS" OR DE "SUPERCROSS"
S25	DE "BICYCLE racing" OR DE "CYCLING competitions" OR DE "STUNT cycling"

S26	motocross or "moto-cross" or supercross or bmx
S27	(track or "off-road" or "freestyl*" or competitive* or terrain or skatepark) N5 (cycling or bicycl* or cyclist* or rider* or racer*)
S28	S24 OR S25 OR S26 OR S27
S29	S23 AND S28
