# Supplementary material for: Injuries, Risk Factors, and Prevention Strategies in Bicycle Motocross (BMX): A Scoping Review
Source: Sports Health. 2024 Oct 26;17(5):965–77. doi: 10.1177/19417381241285037 (PMC11556568; doi:10.1177/19417381241285037)
Supplement: sj-pdf-2-sph-10.1177_19417381241285037 – Supplemental material for Injuries, Risk Factors, and Prevention Strategies in Bicycle Motocross (BMX): A Scoping Review [file sj-pdf-2-sph-10.1177_19417381241285037.pdf]

Appendix 2. List of Included Studies

| First Author | Year | Title |
|---|---|---|
| Aitken | 2014 | Sports-related fractures in South East Scotland: An analysis of 990 fractures |
| Beck | 2016 | Bicycling crash characteristics: An in-depth crash investigation study |
| Black | 2021 | Sport participation and injury rates in high school students: A Canadian survey of 2029 adolescents |
| Brøgger-Jensen | 1990 | Injuries at the BMX Cycling European Championship, 1989 |
| Clarsen | 2021 | Methods for epidemiological studies in competitive cycling: An extension of the IOC consensus statement on methods for recording and reporting of epidemiological data on injury and illness in sport 2020 |
| Diab | 2021 | Pediatric facial fractures in South Australia: Epidemiology, clinical characteristics, and outcomes |
| Durand | 2018 | Traumatic obturator hip dislocation with marginal femoral head fracture in a 15-year-old adolescent: A high-energy trauma - A case report and a review of the literature |
| Engebretsen | 2013 | Sports injuries and illnesses during the London Summer Olympic Games 2012 |
| Fancourt | 2022 | Serious cycling-related fractures in on and off-road accidents: A retrospective analysis in the Australian Capital Territory region |
| Fixsen (Case Report) | 1989 | Bilateral intra-articular loose bodies of the elbow in an adolescent BMX rider |
| Gaskell (Case Report) | 1987 | Prolapsed lumbar intervertebral disc in an adolescent BMX bike rider |
| Goldstein (Abstract) | 2011 | Varied histories of blunt perineal trauma in young men with erectile dysfunction who undergo penile revascularization surgery |
| Hardwick | 2022 | An investigation into helmet use, perceptions of sports-related concussion, and seeking medical care for head injury amongst competitive cyclists. |
| Hurst | 2018 | Profiling of translational and rotational head accelerations in youth BMX with and without neck brace. |
| Illingworth | 1984 | Injuries to children riding BMX bikes |
| Illingworth | 1985 | BMX compared with ordinary bicycle accidents |
| Ipaktchi | 2010 | Subclavian artery and jugular vein rupture after a blunt thoracic trauma due to a BMX handlebar |
| Izumi | 2006 | A case of bilateral testicular calcifications in a bicycle motocross rider accompanied by bulbar urethral injury |

| | | |
|---|---|---|
| Jackson | 1994 | Safety considerations in bicycle moto-cross racing: A case study |
| Johnson | 1987 | Spinal injuries and BMX bicycles |
| Kirchberg (Case Report) | 2021 | Upper thoracic back pain in an Olympic BMX biker |
| Klin | 2016 | Abdominal injuries following bicycle-related blunt abdominal trauma in children. |
| Konczak | 2010 | Chiropractic utilization in BMX athletes at the UCI World Championships: a retrospective study |
| Moyes | 1990 | Injuries to child cyclists in the Bay of Plenty |
| Muthucumaru | 2012 | Trend of severe abdominal injuries from bicycle accidents in children: A preventable condition |
| Oller | 2012 | Recalcitrant supraventricular tachycardia in a professional freestyle BMX rider |
| Park | 1986 | BMX bicycle injuries in children |
| Senturia | 1997 | Bicycle-riding circumstances and injuries in school-aged children |
| Soligard | 2017 | Sports injury an illness incidence in the Rio de Janiero 2016 Olympic Summer Games: A prospective study of 11274 athletes from 207 countries |
| Soligard | 2023 | New sports, COVID-19 and the heat: Sports injuries and illnesses in the Tokyo 2020 Summer Olympics |
| Soysa (Short report) | 1984 | BMX bike injuries: The latest epidemic |
| Sparnon (Case report) | 1982 | BMX handlebar. A threat to manhood? |
| Stathakis (Report) | 1997 | Recreational injury to older children (10-14 year olds) |
| Tierney (Abstract) | 2018 | Effect of BMX riding on head impact kinematics and concussion assessment scores |
| Vaca | 2005 | Skatepark-related injuries in a southern California skatepark and their associated short-term disability and healthcare utilization |
| Wetterhall | 1988 | Injury surveillance at the 1985 National Boy Scout Jamboree |
| Worrell | 1985 | BMX bicycles: Accident comparison with other models |
