# Supplementary figures and images for: Injuries, Risk Factors, and Prevention Strategies in Bicycle Motocross (BMX): A Scoping Review
Source: Sports Health. 2024 Oct 26;17(5):965–77. doi: 10.1177/19417381241285037 (PMC11556568; doi:10.1177/19417381241285037)

Appendix 3. Additional Study Characteristic Figures

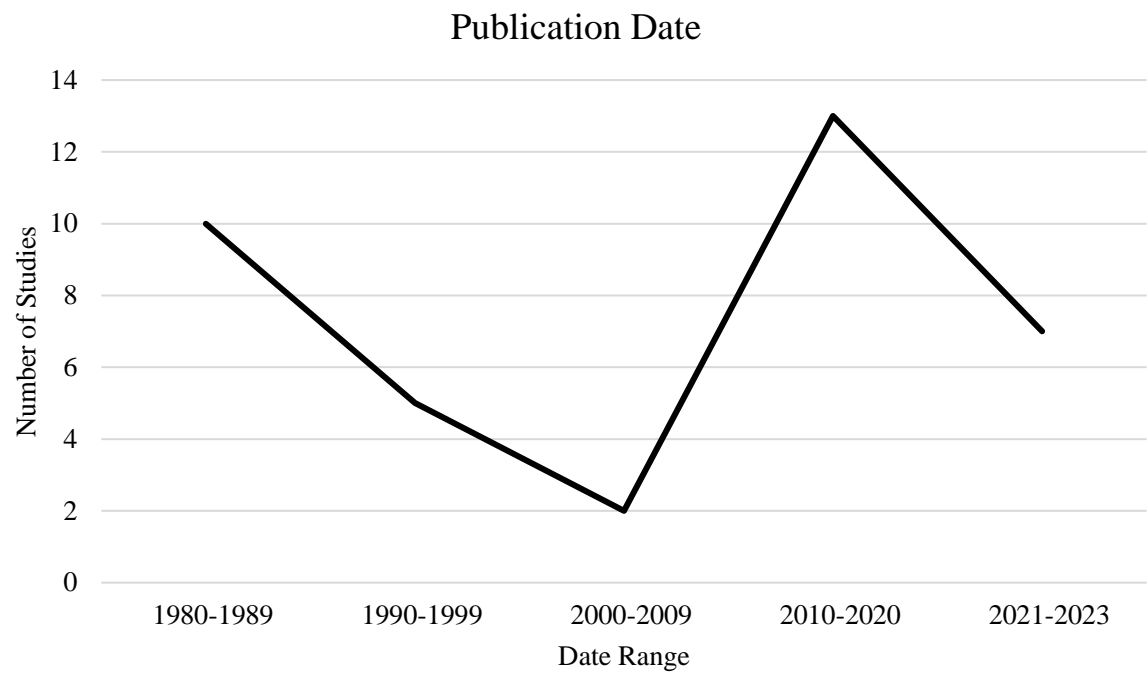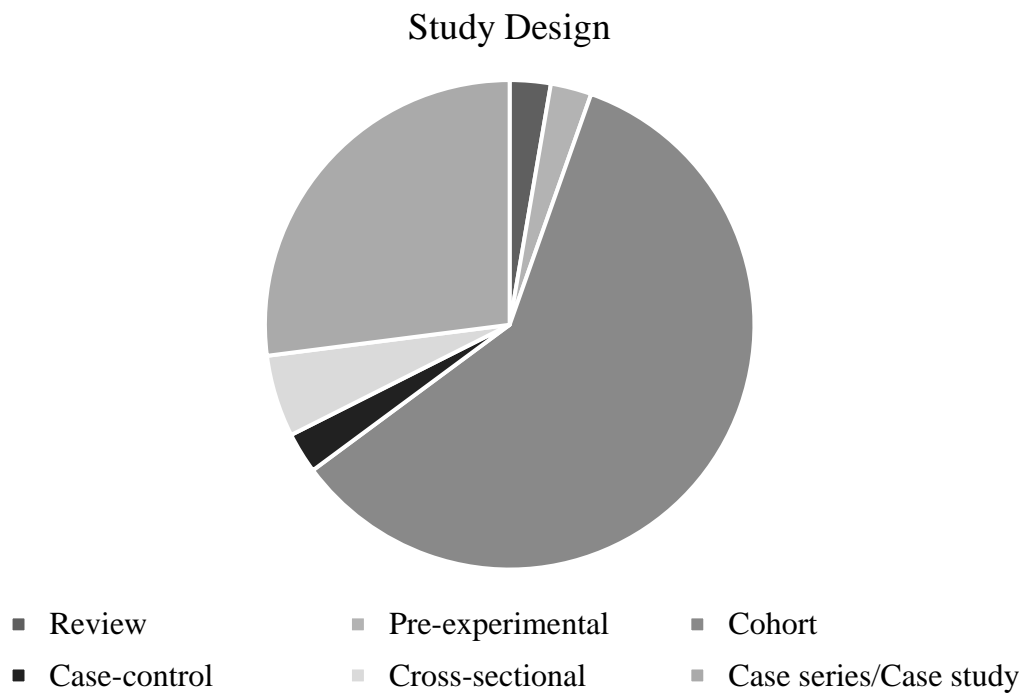

Supplement: sj-pdf-3-sph-10.1177_19417381241285037 – Supplemental material for Injuries, Risk Factors, and Prevention Strategies in Bicycle Motocross (BMX): A Scoping Review [file sj-pdf-3-sph-10.1177_19417381241285037.pdf]
